# Bioinformatic identification of FGF, p38-MAPK, and calcium signalling pathways associated with carcinoma *in situ *in the urinary bladder

**DOI:** 10.1186/1471-2407-8-37

**Published:** 2008-01-31

**Authors:** Malene Herbsleb, Ole F Christensen, Thomas Thykjaer, Carsten Wiuf, Michael Borre, Torben F Ørntoft, Lars Dyrskjøt

**Affiliations:** 1 Molecular Diagnostic Laboratory, Aarhus University Hospital Skejby, Brendstrupgaardsvej 100, DK-8200 Aarhus N, Denmark; 2 Dept. of Genetics and Biotechnology, Faculty of Agricultural Sciences, University of Aarhus, DK-8830 Tjele, Denmark; 3 BiRC – Bioinformatic Research Center, University of Aarhus, Hoegh-Guldbergs Gade 10, Building 1090, DK-8000 Aarhus C, Denmark; 4 Department of Urology, Aarhus University Hospital Skejby, Brendstrupgaardsvej 100, DK-8200 Aarhus N, Denmark

## Abstract

**Background:**

Carcinoma *in situ *(CIS) is believed to be a precursor of invasive bladder cancer. Identification of CIS is a valuable prognostic factor since radical treatment strategies can be offered these patients before the disease becomes invasive.

**Methods:**

We developed a pathway based classifier approach to predict presence or absence of CIS in patients suffering from non muscle invasive bladder cancer. From Ingenuity Pathway Analysis we considered four canonical signalling pathways (p38 MAPK, FGF, Calcium, and cAMP pathways) with most coherent expression of transcription factors (TFs) across samples in a set of twenty-eight non muscle invasive bladder carcinomas. These pathways contained twelve TFs in total. We used the expression of the TFs to predict presence or absence of CIS in a Leave-One-Out Cross Validation classification.

**Results:**

We showed that TF expression levels in three pathways (FGF, p38 MAPK, and calcium signalling) or the expression of the twelve TFs together could be used to predict presence or absence of concomitant CIS. A cluster analysis based on expression of the twelve TFs separated the samples in two main clusters: one branch contained 11 of the 15 patients without concomitant CIS and with the majority of the genes being down regulated; the other branch contained 10 of 13 patients with concomitant CIS, and here genes were mostly up regulated. The expression in the CIS group was comparable to the expression of twenty-three patients suffering from muscle-invasive bladder carcinoma. Finally, we validated our results in an independent test set and found that prediction of CIS status was possible using TF expression of the p38 MAPK pathway.

**Conclusion:**

We conclude that it is possible to use pathway analysis for molecular classification of bladder tumors.

## Background

Carcinoma *in situ *(CIS) is characterized by flat, non-papillary, disordered proliferation and differentiation of urothelial cells. The tumor cells are per definition high grade and are usually associated with significant architectural disorder like loss of polarity and maturation [1]. The cells are highly dysplastic and only weakly adherent. Though CIS is a non-invasive condition progression to the invasive stage is seen in about 50% of the cases [2].

It is believed that bladder tumors develop through at least two distinct genetic pathways: A low grade form and a high grade form often associated with concomitant CIS. The low grade form is frequently associated with loss-of-heterozygocity (LOH) in chromosome 9 and activating mutation of the FGF receptor 3 (*FGFR3*). These tumors often recur but seldom progress to an invasive stage. The high grade form is characterised by *TP53 *gene inactivation and progression to a muscle invasive stage seems to require a subsequent loss of chromosome 9 [3]. CIS is believed to be a precursor of muscle invasive cancers [3-5].

CIS is seldom diagnosed as the primary lesion, since CIS is rarely occurring alone, and may be associated with few symptoms. CIS is usually diagnosed from selected site biopsies taken at cystoscopies, however, the procedure is time-consuming and discomforting to the patients. Therefore, the ability to diagnose CIS before the disease progresses would be extremely valuable and important for establishment of appropriate treatment. Efforts have been made to make such diagnoses using expression profiling. Dyrskjot et al. previously showed that tumors with surrounding CIS have a notably different expression profile than tumors without CIS [6,7] and they were able to construct a 16-gene CIS classifier [6]. Using this gene signature derived from papillary non muscle invasive tumors they could discriminate CIS lesions from normal urothelium samples obtained from individuals with no bladder cancer history. They therefore suggested that for bladder tumors with concomitant CIS, a CIS signature is present in general in the urothelium no matter whether the urothelial cells are organized as tumors, as flat lesions, or as histologically normal-appearing urothelium adjacent to tumor lesions [6]. Furthermore, specific gene expression patterns in tumors with concomitant CIS were found to resemble the gene expression found in muscle invasive tumors. [7]. These findings were confirmed by Wild [8].

The role of FGFR3 mutations in bladder cancer has been intensively studied in the last decade and it has been shown that activating mutations of FGFR3 is related to tumors of low grade and with good prognosis (Reviewed in [9]). Zieger et al found that tumors with concomitant CIS were generally *FGFR3 *wild type [10]. Classification of the tumors using the previously reported CIS classifier [6] showed a strong correlation between tumors classified as "no CIS" and tumors with FGFR3 mutations. The results indicate that the decreasing frequency of FGFR3 mutations in patients with higher tumors stages is caused by the emergence of tumors following a different molecular pathway with no FGFR3 mutations but with presence of CIS.

Previously, molecular classifier approaches for bladder cancer have focused on delineation of best markers. The use of whole pathways for classification has to our knowledge not been investigated. Here, we developed a pathway based classifier approach to predict presence or absence of CIS in patients suffering from non muscle invasive bladder cancer. This is an explorative approach based on the assumption that genes, which, at an individual level, contribute minimally can have explanatory effects when their effects are evaluated together. We computed the pairwise correlations between the expression of transcription factors (TFs) in 37 signalling pathways. From pathways where TFs behaved coherently we used the expression of the TFs to predict presence or absence of concomitant CIS.

## Methods

### Datasets

Three datasets previously described were included in the analysis: 1) A papillary tumor set consisting of material from 28 patients with non muscle invasive bladder tumors: 15 samples from patients with Ta tumors without CIS in selected site biopsies at any visit and 13 patients with Ta or T1 tumors with concomitant CIS. All patients had no history of muscle invasive tumors [6]. The clinical data for these patients is listed in Additional File 1. 2) 9 histologically normal urothelial samples from individuals with prostatic hyperplasia or urinary incontinence and 10 biopsies from 5 cystectomy specimens. Five of the 10 biopsies were CIS lesions and five were histological normal samples located adjacent to CIS. The latter were previously shown to express the CIS signature [6]. 3) Further, data from twenty-three patients suffering from T2-4 muscle invasive bladder tumors were also included. For description of these data, see [11]. All original data are found at [12] with accession numbers GSE3167 and GSE5287.

Informed consent was obtained from all enrolled patients and the protocol was approved by the Scientific Ethical Committee of Aarhus County.

Gene expression was measured using Affymetrix HG-U133A GeneChips with 22,283 probes covering 14,500 genes. Gene expression measures were generated and data normalized using the GCRMA method [13] in ArrayAssist (4.2.0, Stratagene). Hierarchical cluster analysis was conducted using Gene Cluster 2.0 [14] and TreeView 2.0 was used for visualization [15]. The Principal Component Analysis (PCA) was carried out using the PCA function in R.

### Canonical pathways and transcription factors

All canonical signalling pathways in Ingenuity Pathway Analysis (IPA) were analysed to identify if they contained transcriptional regulators. These 43 pathways contained 188 transcriptional regulators and the regulators were studied on the Ingenuity's "Gene View Pages" to ensure that they contained a classical DNA binding domain (zinc finger domains, homeodomain or homeoboxes, helix-loop-helix domains, winged-helix (forkhead) proteins, or leucine-zipper proteins). 116 (62%) TFs had a DNA binding domain and were enrolled for further analysis.

For each pathway a list of TFs was composed, and genes were mapped by Ingenuity using Entrez Gene IDs.

### Transcription factors and downstream targets

The transcription factor's (TF's) downstream targets were identified in Ingenuity by adding the TF to "my pathway page". Using the database all node types, which were linked directly and downstream to the TF through the search-words "activation", "inhibition", "expression", or "transcription", were defined as downstream targets. Lists of the extracted downstream targets were exported with Entrez Gene identifiers. We identified 348 unique downstream targets for the 12 TF's in the 4 significant differentially regulated pathways. After filtering the data the analysis included 219 unique downstream targets.

### Probes, filtering of data, and linking to Entrez Gene Identifiers

Non-unique probes with "_x_at" suffix were excluded. The annotation of probes to genes was based on annotation files from NETAFFX Analysis Center (downloaded 17 August 2006). Due to Unigene Id being less ambiguous than EntrezGene Id, we used Unigene Id for the identification of genes. If a gene contained both "_s_at" and "_at" probes the last were preferred. If more than one probe corresponded to the same UniGene ID the mean expression value was calculated. The outcome was a list of 12,356 genes of which 8,943 were covered by "_at" probes.

To exclude non-varying and non-expressed genes the data were filtered to include only genes where at least 2 samples had a log2-expression above 6, giving 5,562 genes or ~45% of the whole dataset. 59 of the selected 116 TFs fulfilled the filtering criteria. Entrez gene Ids were mapped to UniGene numbers for the 116 selected TFs to link the expression values to the pathways. The 59 TFs and corresponding gene expression data are listed in Additional File 2.

### Scores

Pearson correlations were calculated to determine the correlation between a pair of genes g and h for all samples s:

$$r_{g,h}=\frac{\sum_{s}\left(x_{gs}-{\bar{x}}_{g})(x_{hs}-{\bar{x}}_{h}\right)}{\sqrt{\sum_{s}{\left(x_{gs}-{\bar{x}}_{g}\right)}^{2}}\sqrt{\sum_{s}{\left(x_{hs}-{\bar{x}}_{h}\right)}^{2}}},$$

where x_gs _denotes the expression value for gene g in samples s and ${\bar{\text{x}}}_{\text{g}}$ is the mean of expression values for gene g.

As in Breslin et al. [16] we defined the *Group Correlation Score *(GCS) as the sum of squares of Pearson correlations. Squaring ensured that both positive and negative correlations contributed to the score.

$$GCS=\sum_{g\neq h}r_{g,h}^{2}$$

We computed Pearson correlations for all pairs of TFs in a pathway and defined *Transcription Factor Group Correlation Score *(TF.GCS) as the sum of squares of these correlations. Further, we calculated the correlations between the downstream targets of a specific transcription factor and defined the *Downstream Target Group Correlation Score *(DT.GCS) as the sum of squares of these correlations.

### Permutations and p-values

For each score an equal number of genes were randomly selected and the GCS computed. This was repeated 10,000 times. The significance of the true GCS was determined as the number of permutations having a score higher than the true score, e.g. if 1 out of the 10,000 permutations had a GCS higher than the GCS calculated from the expression values from TFs in a specific pathway the p-value would be 0.0001.

### Leave-One-Out Cross-validations

We used the expression level of TFs from pathways where the TFs correlated coherently to predict presence or absence of CIS. We used a maximum likelihood Leave-One-Out Cross-Validation (LOOCV) classifier approach as previously described [7]. LOOCV was conducted using 1) the TFs for each pathway and 2) using all TFs found in significant pathways together. No selection of best performing genes was applied Fisher's Exact test was used to test whether the predictions were significantly different from the pathological diagnoses of CIS.

## Results

### Co-expression of transcription factors in signalling pathways

First we aimed at identifying active signalling pathways based on the expression level of transcriptional regulators. Therefore, our first issue was to identify pathways where the TFs behaved coherently at the transcriptional level, indicating that the whole pathway was affected. We used the canonical signalling pathways included in IPA and defined the *Transcription Factor Group Correlation Score *(TF.GCS) based on Pearson correlations calculated from expression values of the TFs in the canonical pathways. A high score implies a coherent expression. Clinical information was not included to calculate the score.

We calculated TF.GCS for 37 pathways and based on 10,000 random permutations of the 12,356 genes we computed an associated p-value. p-values were not available from six pathways due to presence of only one TF (see Additional File 3). Four of thirty-seven signalling pathways from IPA showed a p-value < 0.05: FGF signalling pathway (3 TFs), p-value = 0.0021; p38 MAPK signalling (9 TFs), p-value = 0.0021; cAMP signalling pathway (2 TFs), p-value 0.0099; Calcium signalling (5 TFs), p-value = 0.0318 (Table 1). None of the four pathways were significant after Bonferroni correction, however, the 37 available p-values were clearly non-uniformly distributed and showed an overweight of low p-values (mean 0.388; p < 0.005; see Additional File 4), indicating that some of the TF.GCSs are non-random.

**Table 1:** Pathways with significant transcription factor correlations (TF.GCS).

**Pathway**	**Transcription factors in pathways**	**p-value of TF.GCS**
FGF signalling	CREB1, ATF2, STAT3	0.0021
P38 MAPK signalling	CREB1, ATF2, DDIT3, HMGN1, MEF2A, MEF2C, MYC, ATF1, STAT1	0.0021
cAMP signaling	CREB1, STAT3	0.0099
Calcium Signaling	CREB1, CREBBP, MEF2A, MEF2C, HDAC3	0.0318

For these four pathways (with 12 TFs), we also calculated a *Group Correlation Score *between the downstream targets (DTs) of the single TFs. Nine of the 12 TFs had more than one downstream target and for these TFs DT.GCS was computed based on the expression levels of the DTs. p-values were obtained by permutation tests using 10,000 random permutations (see Additional File 5). Only one TF demonstrated a significant correlation between the downstream targets: STAT1 (17 DTs): p-value < 0.0001.

### Classification of tumors based on pathways with coherently expressed transctiption factors

After identifying four pathways with correlated expression between TFs, we investigated if the expression level of these coherently expressed TFs could be used to classify the tumor samples according to CIS diagnoses of the patients. We used a maximum likelihood classifier as previously described [7] to perform Leave-One-Out Cross-Validation (LOOCV) based on both the TFs in each individual pathway and on all 12 TFs. The expression levels of the 12 TFs are listed in Additional File 6. For three of the four pathways we were able to classify the samples according to CIS status significantly: Calcium signalling (Fisher's Exact Test, p = 0.02); FGF signalling (Fisher's Exact Test, p = 0.0004); and p38 MAPK signalling (Fisher's Exact Test, p = 0.0056). Furthermore, all twelve TFs from the four pathways showed classification significantly associated with CIS status (Fisher's Exact Test, p = 0.02). See Additional File 7.

### Gene expression in the FGF and p38 MAPK pathways in CIS versus no-CIS associated tumors

**Figure 1:**
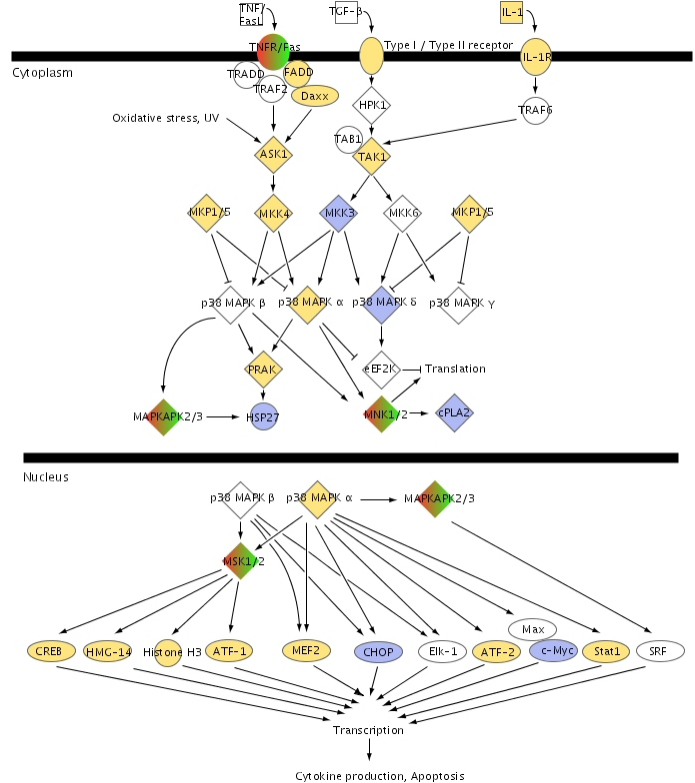
p38 MAPK canonical signalling pathway. Genes are colored according to the log ratio gene expression values between tumors with concomitant CIS and tumors without concomitant CIS. Yellow gene-symbols indicate up-regulation and blue gene symbols indicate down-regulation. The pathway is from the Ingenuity software.

**Figure 2:**
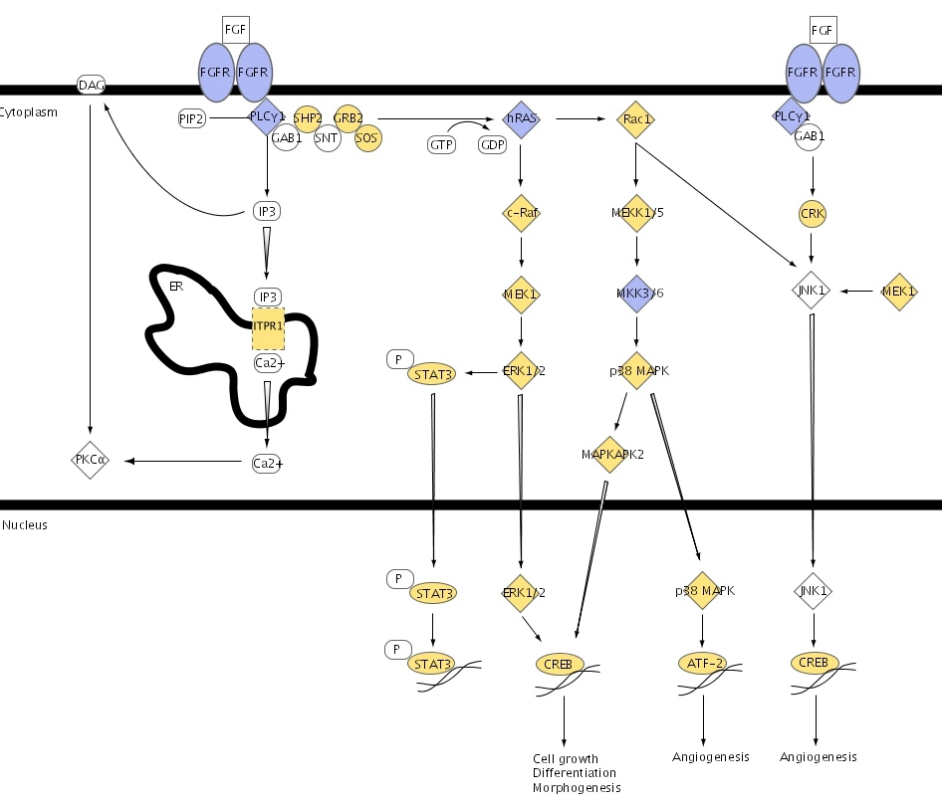
FGF canonical signalling pathway. Genes are colored according to the log ratio gene expression values between tumors with concomitant CIS and tumors without concomitant CIS. Yellow gene-symbols indicate up-regulation and blue gene symbols indicate down-regulation. The pathway is from the Ingenuity software.

We found that the ability to predict CIS status based on the TFs in the P38 MAPK pathway (Figure 1) was most interesting since it contained a relative large number of TFs. Further, it has previously been suggested to have a role in bladder cancer development [9]. Moreover, the FGF pathway (Figure 2) was also interesting because it, among other genes, contained the well described FGF receptor, FGFR3. We calculated the mean log ratios of all expressed genes from the tumors with concomitant CIS compared to the expression in tumors without concomitant CIS and superimposed the gene expression data to the FGF and p38/MAPK canonical pathways. The canonical pathway for p38 MAPK signalling showed that the majority of genes were up regulated in tumors with adjacent CIS compared to tumors without adjacent CIS (Figure 1). For example interleukine 1 (IL-1), which activates the pathway, was found to be up regulated together with its receptor, interleukin 1 receptor beta (IL1R). Further, the kinases mitogen activated kinase kinase kinases, MAP3K5 and MAP3K7 were both up regulated like the kinase-kinase MAP2K4 and the mitogen activated kinase p38 MAPK α. In the nucleus, p38 MAPK α regulates transcription of several targets, and our data showed that in tumors with concomitant CIS the majority of these targets were up regulated, like cAMP responsive element binding protein 1 (CREB1), activating transcription factor 1 and 2 (ATF1, ATF2), MADS box transcription enhancer factor 2, polypeptide A and C (MEF2A, MEF2C), signal transducer and activator of transcription 1 (STAT1), while DNA-damage-inducible transcript 3 (DDIT3 = CHOP) was down regulated.

The canonical pathway for FGF signalling showed likewise an up regulation of the majority of the genes including some of the kinases from the MAPK signalling like MAPK1, MAP2K1 and MAP3K5. Likewise, signal transducer and activator of transcription 3 (STAT3) and p38 MAPK (MAPK14) were up regulated while FGFR3 was down regulated in tumors with concomitant CIS compared to tumors without concomitant CIS (See Figure 2).

### PCA and hierarchical cluster analysis

**Figure 3:**
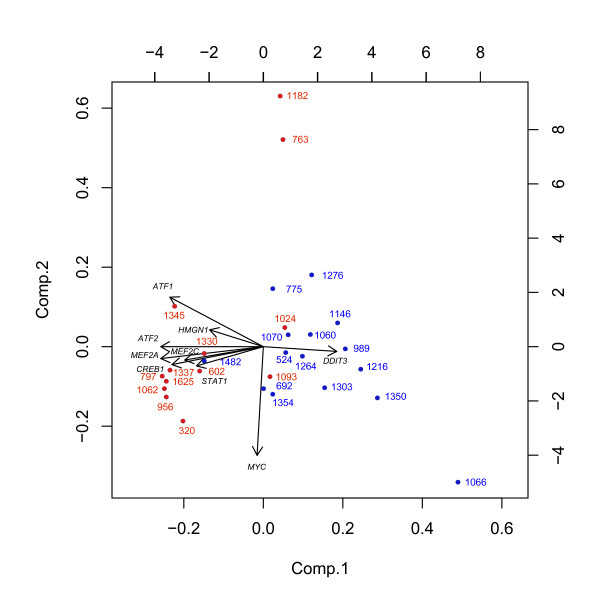
Biplot of the first two principal components from the Principal component analysis of the p38 MAPK signalling pathway. Tumors with concomitant CIS are indicated with red numbers. Tumors with no concomitant CIS are indicated with blue numbers.

Since the P38 MAPK pathway contained most of the 12 TFs we continued our analysis focusing on this pathway. To visualize the expression of the different TFs in this pathway a PCA was made and a biplot constructed. On the biplot, colors were superimposed to show the two groups of tumors (tumors with or without concomitant CIS). The biplot shows the first two principal components from the PCA of the p38 MAPK signalling pathway (Figure 3).

The first principal component (x axis) explains 48% of the variation and is positively correlated with DDIT3, uncorrelated with MYC and negatively correlated with the main group of genes, i.e. all other genes. The second principal component (y axis) explains 13% of the variation and is negatively correlated with MYC and not highly correlated with the other genes.

Patients located on the left of the plot have a low first principal component, and hence a high expression for the main group of genes and a negative expression for DDIT3, whereas the opposite is the case for patients located on the right of the plot. Patients 1182 and 763 is located on the upper part of the plot and therefore have a high value of the second principal component, i.e. a low expression of MYC.

By superimposing colours on the plot we show that tumors with concomitant CIS are located on the left of the plot, and therefore have the main group of genes up regulated and DDIT3 down regulated. The plot indicates that the two tumors with concomitant CIS from patients 1182 and 763 may be a separate group with MYC down regulated.

The PCA biplot for the p38 MAPK pathway also visualises that expression for most of the genes apart from MYC and DDIT3 is positively correlated, while being negatively correlated with DDIT3. MYC is nearly uncorrelated with all other genes in the pathway.

**Figure 4:**
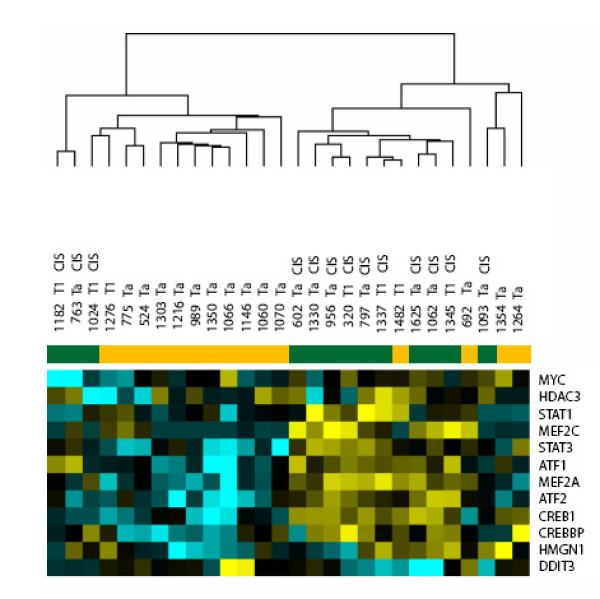
Cluster analysis of 28 bladder cancer patients (columns) based on the gene expression of the twelve transcription factors found in the four pathways with significantly correlating transcription factors (rows). Up regulated genes from tumors without concomitant CIS to tumors with concomitant CIS are coloured yellow while down regulated genes are blue. The colour bar indicates the clinical diagnosis, green, concomitant CIS; orange, no concomitant CIS.

To analyze this further, we conducted a cluster analysis of the gene expression of the twelve TFs from the four pathways (see Figure 4). One cluster branch contained mainly down regulated genes. Here we found 3 CIS patients and 11 no CIS patient. The second cluster branch contained 10 patients with CIS and four without CIS (Fisher's Exact test, p-value = 0.0213).

### Pathway regulation in muscle invasive tumors

**Figure 5:**
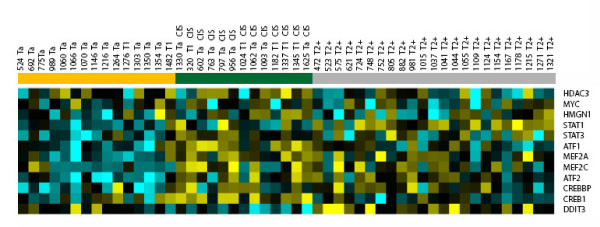
Heatmap illustrating the expression level of the twelve transcription factors (rows) found in the four signalling pathways: FGF, p38 MAPK, cAMP and calcium. The columns contain expression of the 15 patients without CIS, 13 patients with adjacent CIS and 23 patients with muscle invasive bladder carcinoma, mTCC. Up regulated genes are yellow, down regulated genes are blue, while black indicates no change with no CIS samples as reference. The colour bar indicates the clinical diagnosis, green, CIS; orange, no CIS, gray, muscle invasive tumors.

We next investigated whether the pathway regulations identified in the tumors with concomitant CIS were comparable to the pathway regulations identified in patients with muscle invasive bladder tumors. Figure 5 shows the expression of the twelve TFs in tumors with and without concomitant CIS and in muscle-invasive tumours (23 patients). The expression of seven of the twelve genes was significantly different when comparing the non muscle invasive tumors without concomitant CIS to both the tumor with concomitant CIS and to the muscle invasive tumors (t-test, *ATF2*, p-value = 1.2E-4; *CREBBP*, p-value = 1.2E-2; *MEF2A*, p-value = 1.7E-4; *MEF2C*, p-value = 1.7E-3; *ATF1*, p-value = 2.6E-2; *STAT1*, p-value = 1.3E-4; *STAT3*, p-value = 1.2E-2). A hierarchical cluster analysis based on the seven genes significantly separated the patients according to the groups no CIS and CIS/Invasive (χ^2^-test, p < 2.0E-5). This result indicated that the tumors with concomitant CIS showed an expression similar to the expression found in muscle invasive tumors for the majority of the twelve selected TFs. This is comparable to the large similarities previously found [6].

### Pathway based classification using normal and CIS samples

**Figure 6:**
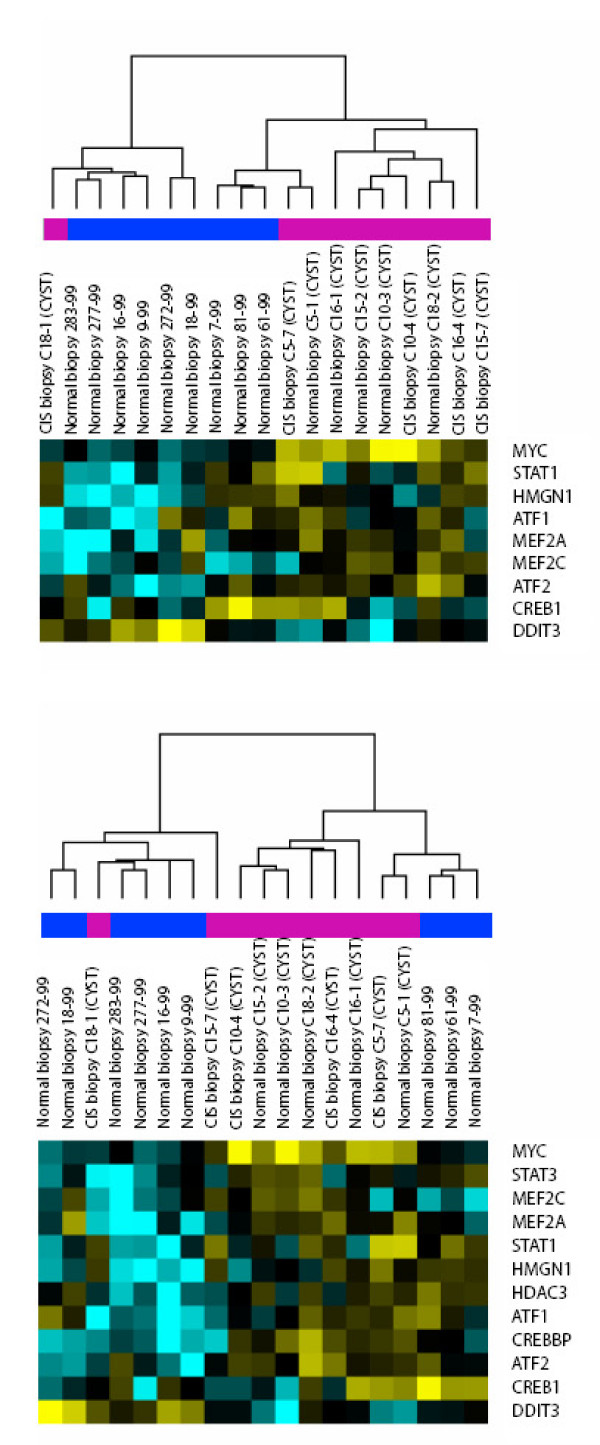
Cluster analysis of 9 normal samples and 10 samples from cystectomies with CIS. Top: TFs from p38 MAPK signalling pathway. Bottom: All 12 TFs used for clustering. The color bar indicates the clinical diagnosis, blue, histologically normal urothelial samples; pink, cystectomy specimens.

After identification of pathways with relevance for identification of concomitant CIS in non muscle invasive bladder carcinomas we investigated if these pathways were also important in actual CIS lesions. For this we used a set of nine urothelial samples from healthy individuals and ten biopsies from cystectomy specimens (five were CIS lesions and five were histological normal samples located adjacent to CIS). The five histologically normal samples located adjacent to CIS were previously shown to express the CIS signature [6] and this set was previously used to validate the CIS classifier developed by Dyrskjøt et al. [6] We used a hierarchical cluster analysis approach for classification of the samples (see Additional File 8). The p38 MAPK signalling pathway showed a significant correlation to CIS status for the patients in these independent test samples (p = 0.0198), while the other pathways showed no significant correlation to CIS status in this small sample set. The TFs from the four pathways combined did not show a statistically significant correlation to CIS status, although the p-value was borderline (p = 0.0698; Figure 6). Like in the papillary tumors, the cluster analysis showed that the expression of the majority of the twelve genes was down regulated in normal biopsies compared to the ten biopsies from cystectomy specimens. The biopsies from the cystectomy specimens clustered together, and this was independent of whether the specimen was a CIS lesion or a histological normal sample located adjacent to CIS, underscoring the tight relation between CIS-and "normal" looking biopsies in this urothelium. DDIT3 was up-regulated in the normal samples compared to the biopsies from cystectomy specimens, while MYC was up regulated in the cystectomy specimens but not differentially expressed in the papillary tumors.

## Discussion

Several studies have focused on construction of microarray based classifiers for prediction of various aspects of bladder cancer like stage [7,8,11,17,18], progression [7,8,19], recurrence [7], survival [18,20], and treatment response [21]. These classifiers consist of up till a few hundred genes, which not necessarily are associated with the same molecular pathways. Many of these lists have been validated in independent test sets but little overlap has been observed between classifiers identified by different groups, addressing the same issue. Our study differs from the traditional classifier approach since we were interested in identifying whole pathways, which are associated with a distinct disease course. A comprehensive understanding of the underlying molecular networks involved in disease development and progression is necessary for development of new treatments and may also be useful in a molecular diagnostic approach as a supplement to the common pathological classification.

Here, we used a pathway based classifier approach to predict a clinical parameter in patients suffering from non muscle invasive bladder cancer. We used IPA in a hypothesis generative manner to suggest which pathways are most relevant for detection of concomitant CIS. IPA is a relatively new bioinformatics tool, which enables the user to get information of relationships between genes of interest and about functions, diseases, and drugs, related to these genes. IPA contains lists of metabolic and signalling pathways. These pathways are termed canonical pathways, since they contain well-established knowledge about specific relationships between groups of genes. The challenge related to the use of these networks is that it is arbitrary to define where a network starts and ends. Moreover, it should be noted that the information in the IPA knowledge base is gathered from a broad variety of conditions and from three different organisms (human, mouse, and rat). Further, regulation is affected on several levels: the transcriptional level and the post-transcriptional level, where phosphorylations, glucosylations and various other epigenetic events contribute.

The advantage of a network based strategy is that pathways can include small contributions from single genes. Alone these genes might not seem to have an effect and would not have been detected in a single-gene analysis strategy. Together, these small contributions may have a great impact on the malignant development. The importance of an orchestrated effect has previously been shown in a excellent study of breast cancer [22]. Further, using such an additive strategy implies that the risk of false positives may be minimized since effects of single genes must be in consensus with the effects from other genes in the pathway before they will be ascribed any importance.

We investigated the pairwise correlations between TFs in 37 signalling pathways from IPA and calculated the sum of squared correlations across samples in a set of twenty-eight non muscle invasive bladder carcinomas. We identified four signalling pathways (p38 MAPK, FGF, calcium and cAMP containing twelve TFs in total) where the expression of TFs seemed to behave coherently. TF expression levels in three of these pathways (FGF, p38 MAPK, and calcium signalling) or the expression of the twelve TFs together could be used to predict presence or absence of concomitant CIS. Comparing the non muscle invasive samples with twenty-three patients suffering from muscle-invasive bladder carcinoma showed that the majority of the TFs' expression in non muscle invasive samples without concomitant CIS was significantly different from the expression in non muscle invasive tumors with adjacent CIS and in muscle invasive tumors. This is consistent with previous results indicating that CIS is the precursor for invasive disease [3]

Our study indicates that the expression of genes involved in p38 MAPK signalling pathway is up regulated in tumors with concomitant CIS. p38 is a mitogen activated protein kinase (MAPK) and is part of one of three distinct MAPK pathways, which also include the ERK1 and 2 pathways and the JNK1 pathway. These pathways are signalling cascades involving at least three levels of MAP kinases. The p38 MAPKs respond to environmental stress like ultraviolet light, heat, osmotic shock, and inflammatory cytokines. The signal activates the serine/threonine kinases termed MAPKKK, which phosphorylate the MAPK kinases (MAPKK). The MAPKKs then activate the p38 MAPK kinases by dual phosphorylation of specific threonine and tyrosine residues. Activated p38 MAPK can phosphorylate several substrates like TFs, other kinases and cytosolic proteins. Some of these components are responsible for inflammatory cytokine production and a great effort has been done to try to develop inhibitors of p38 MAPK to prevent inflammatory diseases like rheumatoid arthritis [23].

Mutations of the FGF receptor 3 gene (*FGFR3*) have been shown to be a distinct characteristic of tumors with low grade and stage. Up to 80% of low-grade Ta tumors show the mutations, which constitutively activate the receptor. It has been suggested that the consequences of FGFR3 activation in the urothelium is activation of the MAPK and/or the PI3-kinase pathway [9,24]. Our results highlight the already known relation between the FGF and the p38 MAPK pathways. The FGF pathway contains members of the mitogen-activated protein kinase family like MAP2K3, MAP2K6, MAP3K5, MAPK14, MAPKAPK2, and p38 MAPK (See Figure 1 and Figure 2). Constitutive activation of FGFR3 will probably cause constitutive activation of p38 MAPK and thereby activate the entire p38 MAPK signalling pathway. Interestingly, in the papillary tumors we found that the expression level of TFs from the FGF pathway could be used to predict presence or absence of concomitant CIS. However, the FGF pathway does not seem to play such a clear role in the CIS lesions themselves, as these could not be identified based on FGF expression alone. Whether this is due to cells being lost by exfoliation, so we do not measure the right cells, or a regulatory process is overruling the FGF signalling in the CIS lesions, which among other parameters are surrounded by massive inflammation, is at present unknown.

In a study conducted by Breslin et al. [16] a pathway approach was used on datasets from breast cancer [25,26] and leukemia [27]. In that study expression values of downstream targets were included. We tried to include downstream targets in our analysis and identified downstream targets for the 12 TFs using direct downstream relations known by the Ingenuity Pathway Knowledge Base (IPKB). However, only one of nine TF showed a significant GCS for the downstream targets. We believe that the information gathered in the IPKB is too unspecific to identify downstream targets in an automatic manner. Identification of TF binding motifs followed by bioinformatics screenings of possible binding sites would be necessary to predict downstream targets more accurately.

## Conclusion

In conclusion, we have shown that twelve TFs from four signalling pathways can be used to predict presence of concomitant CIS and especially TFs from the p38 MAPK signalling pathway seem to be important. Other pathways may be of importance but could not be delineated in this relative limited sample set. The approach is convincing as it shows how complex multiparameter data sets can be subjected to bioinformatic analyses and pin-point pathways that may later be targets for therapeutic intervention. Furthermore, it would be interesting to use a similar strategy to investigate the pathways involved in invasive and metastatic disease courses.

## Abbreviations

CIS (carcinoma in situ); TF (Transcription factor); IPKB (Ingenuity Pathway Knowledge Base); IPA (Ingenuity pathway analysis); TF.GCS (Transcription Factor Group Correlation Score); LOOCV (Leave-One-Out Cross-Validation); GCS (Group Correlation Score)

## Competing interests

The author(s) declare that they have no competing interests.

## Authors' contributions

MH, OFC, TT, CW, MB, TFØ and LD participated in the study design. MH and OFC performed most of the microarray analyses. LD performed cluster and classification analyses. MH drafted the paper. MH, OFC, TFØ and LD participated in the final preparation of the paper. All authors read and approved the final manuscript.

## Pre-publication history

The pre-publication history for this paper can be accessed here:



## Supplementary Material

Additional file 1 Clinical information. Clinical information for patients with non-muscle invasive tumors Click here for file

Additional file 2 Transcription factors. 59 Transcription factors and corresponding gene expression levels Click here for file

Additional file 3 TF.GCS. Table with 37 pathways and corresponding TF.GCS and p-values Click here for file

Additional file 4 TF.GCS distribution. Distributions of group correlation scores for transcription factors in 37 signalling pathways Click here for file

Additional file 5 TF.GCS p-values. p-values for group correlation scores of transcription factors' downstream targets (DT.GCSs) Click here for file

Additional file 6 Expression levels. 12 TFs and corresponding gene expression levels Click here for file

Additional file 7 Classification. Contingency table of the clinical diagnosis compared with the prediction of CIS/no CIS based on Leave-One-Out Cross-Validation using expression values of pathway specific transcription factors Click here for file

Additional file 8 Validation. Independent test set validation results. Cluster analysis using transcription factors in 4 pathways was used to classify the samples Click here for file
