# Supplementary material for: Bioinformatic identification of FGF, p38-MAPK, and calcium signalling pathways associated with carcinoma in situ in the urinary bladder
Source: BMC Cancer. 2008 Jan 31;8:37. doi: 10.1186/1471-2407-8-37 (PMC2268699; doi:10.1186/1471-2407-8-37)
Supplement: Additional file 3 — TF.GCS. Table with 37 pathways and corresponding TF.GCS and p-values [file 1471-2407-8-37-S3.doc]

Group correlation scores for transcription factors in 43 signalling pathways. Number of transcription factors for each pathway is listed. NA: not available.
