# Supplementary material for: Bioinformatic identification of FGF, p38-MAPK, and calcium signalling pathways associated with carcinoma in situ in the urinary bladder
Source: BMC Cancer. 2008 Jan 31;8:37. doi: 10.1186/1471-2407-8-37 (PMC2268699; doi:10.1186/1471-2407-8-37)
Supplement: Additional file 5 — TF.GCS p-values. p-values for group correlation scores of transcription factors' downstream targets (DT.GCSs) [file 1471-2407-8-37-S5.doc]

p-values for group correlation scores of transcription factors’ downstream targets (DT.GCSs).
