# Supplementary material for: Bioinformatic identification of FGF, p38-MAPK, and calcium signalling pathways associated with carcinoma in situ in the urinary bladder
Source: BMC Cancer. 2008 Jan 31;8:37. doi: 10.1186/1471-2407-8-37 (PMC2268699; doi:10.1186/1471-2407-8-37)
Supplement: Additional file 7 — Classification. Contingency table of the clinical diagnosis compared with the prediction of CIS/no CIS based on Leave-One-Out Cross-Validation using expression values of pathway specific transcription factors [file 1471-2407-8-37-S7.doc]

Contingency table of the clinical diagnosis compared with the prediction of CIS/no CIS based on Leave-One-Out Cross-Validation using expression values of pathway specific transcription factors.
