# Supplementary material for: Bioinformatic identification of FGF, p38-MAPK, and calcium signalling pathways associated with carcinoma in situ in the urinary bladder
Source: BMC Cancer. 2008 Jan 31;8:37. doi: 10.1186/1471-2407-8-37 (PMC2268699; doi:10.1186/1471-2407-8-37)
Supplement: Additional file 8 — Validation. Independent test set validation results. Cluster analysis using transcription factors in 4 pathways was used to classify the samples [file 1471-2407-8-37-S8.doc]

Validation in an independent test set. Cluster analysis using transcription factors in 4pathways. Fisher’s Exact Test with p-values.
