# Supplementary material for: Bioinformatic identification of FGF, p38-MAPK, and calcium signalling pathways associated with carcinoma in situ in the urinary bladder
Source: BMC Cancer. 2008 Jan 31;8:37. doi: 10.1186/1471-2407-8-37 (PMC2268699; doi:10.1186/1471-2407-8-37)
Supplement: Supplementary file 8 [file 1471-2407-8-37-S4.doc]

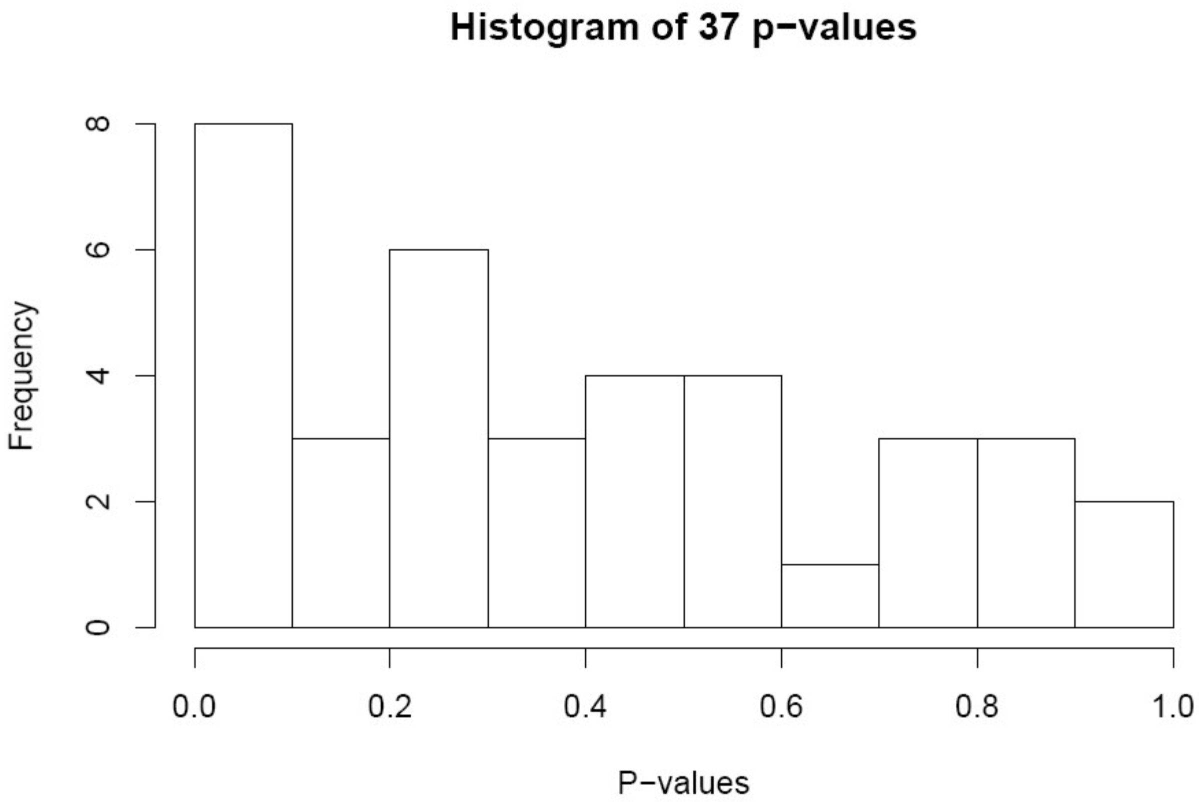
Histogram of 37 p−values
Frequency
0
2
4
6
8
0.0
0.2
0.4
0.6
0.8
1.0
P−values
